# Neurenteric cysts of the spine

**DOI:** 10.4103/0974-8237.65484

**Published:** 2010

**Authors:** Jesse J. Savage, James N. Casey, Ian T. McNeill, Jonathan H. Sherman

**Affiliations:** Department of Neurological Surgery, University of Virginia, Charlottesville, VA, USA; 1 University of Virginia School of Medicine, Charlottesville, VA, USA

**Keywords:** Craniovertebral junction, enterogenous cyst, intraspinal cyst, neurenteric cyst, spinal cord tumor, spinal dysraphism

## Abstract

Neurenteric cysts account for 0.7-1.3% of spinal axis tumors. These rare lesions result from the inappropriate partitioning of the embryonic notochordal plate and presumptive endoderm during the third week of human development. Heterotopic rests of epithelium reminiscent of gastrointestinal and respiratory tissue lead to eventual formation of compressive cystic lesions of the pediatric and adult spine. Histopathological analysis of neurenteric tissue reveals a highly characteristic structure of columnar or cuboidal epithelium with or without cilia and mucus globules. Patients with symptomatic neurenteric cysts typically present in the second and third decades of life with size-dependent myelopathic and/or radicular signs. Magnetic resonance imaging and computed tomography are essential diagnostic tools for the delineation of cyst form and overlying osseous architecture. A variety of approaches have been employed in the treatment of neurenteric cysts each with a goal of total surgical resection. Although long-term outcome analyses are limited, data available indicate that surgical intervention in the case of neurenteric cysts results in a high frequency of resolution of neurological deficit with minimal morbidity. However, recurrence rates as high as 37% have been reported with incomplete resection secondary to factors such as cyst adhesion to surrounding structure and unclear dissection planes. Here we present a systematic review of English language literature from January 1966 to December 2009 utilizing MEDLINE with the following search terminology: neurenteric cyst, enterogenous cyst, spinal cord tumor, spinal dysraphism, intraspinal cyst, intramedullary cyst, and intradural cyst. In addition, the references of publications returned from the MEDLINE search criteria were surveyed in order to examine other pertinent reports.

## INTRODUCTION

The neurenteric cyst is a rare lesion of the spinal axis composed of heterotopic endodermal tissue. During the third week of human embryogenesis, the neurenteric canal unites the yolk sac and the amniotic cavity as it traverses the primitive notochordal plate. Persistence of the normally transient neurenteric canal prevents appropriate separation of endoderm and notochord. This anomalous union manifests as congenital abnormalities of the spine defined by the presence of mucus-secreting epithelium reminiscent of the gastrointestinal tract. These lesions were first described by Kubie and Fulton in 1928 as *teratomatous cysts* and later by Puusepp in 1934 as *intestinomas*, Holcomb and Matson coined the term neurenteric cyst in 1954.[1‐3] Neurenteric cysts account for 0.7-1.3% of all spinal cord tumors.[4] Approximately 90% of neurenteric cysts are located in the intradural/extramedullary compartment, while the remaining 10% are divided between an intradural/intramedullary or extradural location.[5] The majority of literature describing neurenteric cysts is in the form of case reports and case series. We performed a systematic search of English language literature from January 1966 to December 2009 utilizing MEDLINE with the following search terms: neurenteric cyst, enterogenous cyst, spinal cord tumor, spinal dysraphism, intraspinal cyst, intramedullary cyst, and intradural cyst. In addition, the references of reports returned from the MEDLINE search criteria were surveyed in order to examine other pertinent publications. Here we present a concise and up to date review of these intriguing congenital abnormalities of the craniovertebral junction and spine.

## HISTOPATHOLOGY AND PATHOGENESIS

**Figure 1:**
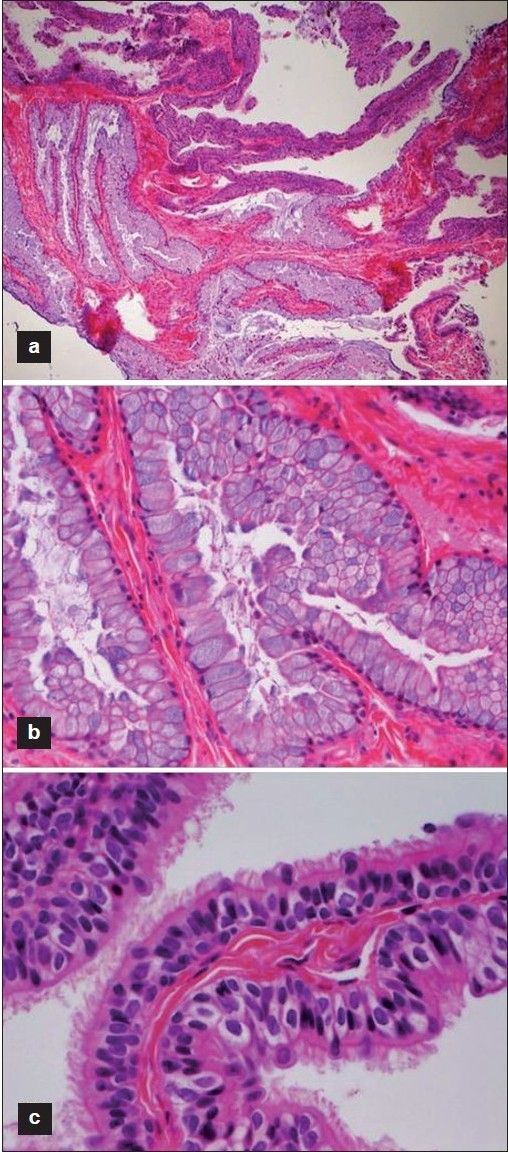
Photomicrograph of neurenteric cyst epithelium. (a) Neurenteric cyst wall (Type A) demonstrating simple columnar and cuboidal epithelium resting upon basal lamina composed of Type IV collagen (H and E, ×200). (b) High-magnification photomicrograph of columnar and cuboidal mucin-secreting goblet cells (H and E, ×1000). (c) High-magnification photomicrograph of ciliated pseudostratified columnar epithelium of neurenteric cyst wall (H and E, ×1000).

The diagnostic histopathology of neurenteric cysts has been described classically on hematoxylin and eosin (H and E) stained samples as a collection of mucin producing simple columnar or cuboidal ciliated and nonciliated goblet cells surrounding a central cystic cavity [Figure 1]. Wilkins and Odom formulated a system to classify neurenteric cysts based upon three histopathological presentations [Table 1].[6] Type A cysts contain either columnar or cuboidal cells, with ciliated and nonciliated components atop a basal membrane composed of type IV collagen. Type B cysts include all of the features of type A as well as additional tissue that may include bone, cartilage, lymphatic tissue, fat, or glandular components. Type C cysts are identified by type A features in association with ependymal or glial tissue. While this classification scheme has been used to categorize histological subtypes, there appears to be no association between Wilkins and Odom subtypes and the site, extent, or outcome after resection of the neurenteric cyst.[6]

**Table 1:** Wilkins and Odom neurenteric cyst histopathological classification system[6]

Characteristics	Type A	Type B	Type C
Single layer of pseudostratified columnar or cuboidal cells mimicking respiratory or gastrointestinal epithelium	+	+	+
Complex invaginations with glandular organization; mucinous or serous production; nerve ganglion, lymphoid, skeletal muscle, smooth muscle, fat, cartilage, and/or bone elements	-	+	-
Ependymal or glial tissue	-	-	+

The cystic epithelial cells typically stain negatively for glial fibrillary acidic protein (GFAP) and positively for cytokeratin, epithelial membrane antigen, and carcinoembryonic antigen (CEA) on immunohistochemical assays. Positive CEA staining supports a theory of shared lineage between the cystic wall and the intestinal mucosa. In the case of intramedullary lesions, astrocytes within the cyst wall may stain positively for GFAP in comparison to the typical negative staining pattern of extramedullary cysts.[5] Confirmation of mucinous production can be established by histochemical assays utilizing mucicarmine, alcian blue, and periodic acid-Schiff (PAS). On gross appearance, neurenteric cysts consist of a thickened outer membrane that surrounds a fluid collection described as straw-colored. Variations on fluid composition include appearances described as "milky," "blackish," "CSF-like," and "clear jelly."[5,7‐9]

The mucus-secreting columnar epithelium of the neurenteric cyst wall has been described to be similar to that of the intestinal or respiratory epithelium.[10,11] Despite the similarities, these lesions often contain ciliated epithelial cells; ciliated epithelia does not exist in the adult gastrointestinal tract. Langman postulated that when the respiratory primordium buds into the ventral foregut in the third week of development, ciliated cells may be displaced toward the evolving ventral spinal cord and result in neurenteric cyst formation.[10] Equally, Fabinyi and Adams emphasized that in the early stages of development, there is ciliated epithelium within the immature esophagus that may provide an origin for the heterotopic ciliated tissue found within the neurenteric cyst.[12] In short, the cellular milieu of the neurenteric cyst is indicative of the embryological time frame in which the defect occurred during development.

The overwhelming majority of neurenteric cysts occur as solitary lesions. However, patients have been reported to present with disseminated disease along the spinal axis. Most of these cases demonstrate local propagation of neurenteric cysts in proximity to the original lesion.[13‐18] Yasuda *et al* reported a case in which a neurenteric cyst treated with surgical fenestration resulted in widespread spinal dissemination noted 9 years postoperatively.[18] Several proposed mechanisms of dissemination have been theorized ranging from activation of quiescent nests to dispersal secondary to incomplete surgical resection. In addition to dissemination, other rare complications associated with neurenteric cysts have been reported. Abhishek *et al* presented a case of an 18-year-old male with sudden neurological deterioration resulting from a *Staphylococcus aureus* infected neurenteric cyst.[19] Additional rare manifestations of neurenteric cysts reported in the literature include spontaneous hemorrhage as well as malignant transformation.[16,20]

## CLINICAL PRESENTATION

Individuals diagnosed with neurenteric cysts most frequently present in the second and third decades of life with a male-to-female ratio of approximately 2:1.[21‐23] Moreover, in the pediatric population, 61.2% of patients found to have neurenteric cysts are male with a mean age of 6.4 years at presentation.[24] The majority of adult patients with neurenteric cysts present with progressive focal pain at the level of spinal axis pathology, fluctuating myelopathic signs, or radicular symptoms. The myelopathic symptoms are typically associated with lesions in the cervical and thoracic spine, while radicular symptoms can present with lesions in either the cervical or lumbar spine. The latter symptoms include focal weakness, radicular pain, or paresthesias depending on the size and location of the lesion. The fluctuating nature of these symptoms have been attributed to cyst volumetric flux associated with periodic leakage of fluid content secondary to osmotic and hemodynamic factors.[25]

The waxing and waning nature of symptoms associated with spinal cord compression secondary to cyst volume instability has frequently led to misdiagnoses of central nervous system (CNS) demyelinating disease.[26] In addition to the more common signs or symptoms, a variety of other clinical manifestations have been noted in the pediatric population. Case reports of pediatric patients have described signs and symptoms that include aseptic meningitis, pyogenic meningitis, chronic pyrexia, incontinence, and paraplegia.[24,25,27‐29]

Neurenteric cysts are allied with bony abnormalities of the spine in approximately 50% of cases and are associated with a variety of conditions including spinal dysraphism, scoliosis, spina bifida, split cord malformation, and Kippel-Fiel syndrome.[30‐36] In addition to disruption of the normal osseous architecture of the spine, neurenteric cysts can be associated with malformations of the gastrointestinal tract, anal atresia, renal defects, cardiac abnormalities, and overlying cutaneous changes.[24]

## DIAGNOSTIC IMAGING

Patients with signs or symptoms associated with neurenteric cysts require a diagnostic evaluation with such imaging modalities as magnetic resonance imaging (MRI) and/or computed tomography (CT). MRI has proven superior in the delineation of the cyst form and its relationship with surrounding neural structures when compared to CT.[30,37,38] Furthermore, utilization of MRI avoids the potentially confounding bony artifact often associated with CT when surveying cyst margins. Nevertheless, the CT scan maintains an essential role in the evaluation of neurenteric cysts secondary to the osseous malformations associated with these lesions. In addition, the CT myelogram can demonstrate a positive meniscus sign. This imaging finding results from the partial dye obstruction in the case of intradural/extramedullary cysts and complete contrast obstruction with intradural/intramedullary cysts.[25]

**Figure 2:**
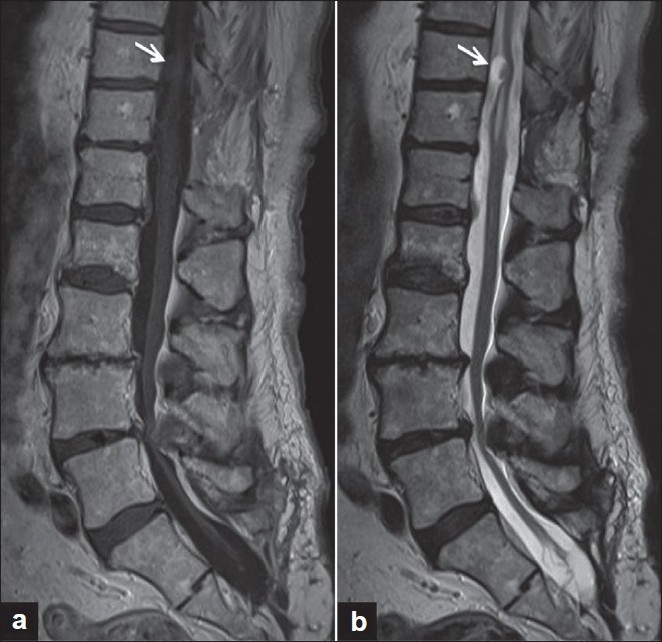
MRI of thoracolumbar junction with intradural/ extramedullary neurenteric cyst at T11. (a) Midsagittal MRI demonstrates isointense lesion (arrow) on T1-weighted and (b) hyperintense signal (arrow) on T2-weighted

The most common MRI findings associated with neurenteric cysts are noncontrast-enhancing lesions that are isointense on T1-weighted sequences and hyperintense on T2-weighted imaging [Figure 2].[11,13,29,39] While these diagnostic results are considered typical of neurenteric cysts, frequent variations are reported. Muzumdar *et al* have described a neurenteric cyst with an abscess or granulomatous-like presentation associated with peripheral enhancement of the cystic structure on MRI.[40] Miyagi *et al* and Nagi *et al* reported cases in which the neurenteric cyst appeared hypointense on T1-weighted imaging and hyperintense on T2-weighted imaging.[39,41] In a two-patient series, Sasani *et al* described one patient with a lesion hyperintense on T1-weighted imaging and hypointense on T2-weighted imaging, while the second patient presented with a cyst hypointense on T1-weighted imaging and hyperintense on T2-weighted imaging.[11] In a series of 18 patients with neurenteric cysts, Kimura *et al* described 16 of 18 lesions hyperintense on T1-weighted imaging with only two cases demonstrating "standard" isointensity on T1-weighted sequences.[13] An important aspect of the Kimura study was the utilization of fluid-attenuated inversion recovery (FLAIR) imaging. All patients in the Kimura study were hyperintense in comparison to cerebrospinal fluid on FLAIR sequences. Utilization of the FLAIR sequence may show increased sensitivity for characterizing neurenteric cysts, especially in combination with other MRI modalities. As the results above detail, variation in MRI findings is frequent and atypical imaging characteristics should not exclude the potential diagnosis of neurenteric cysts.

**Figure 3:**
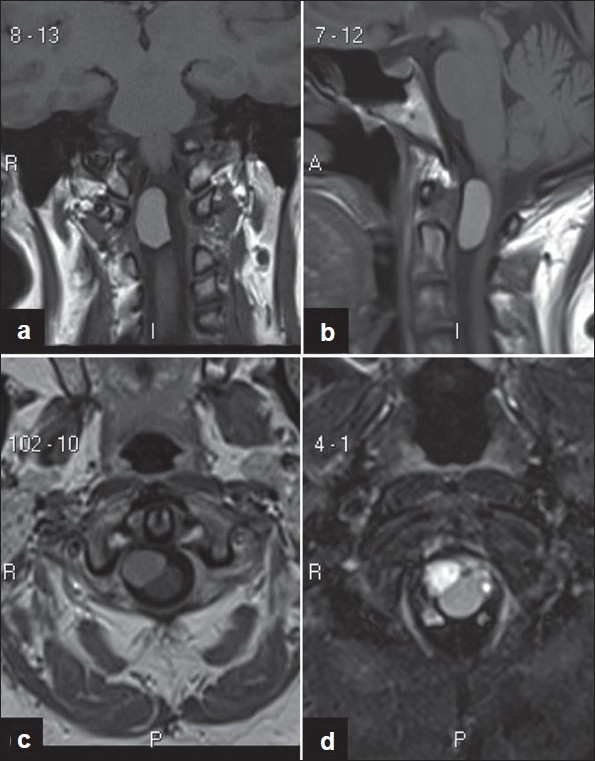
MRI of an intradural/extramedullary neurenteric cyst located at the craniocervical junction. Coronal (a) and sagittal (b) T1-weighted MRI showing the ventrally located homogenous lesion compressing the cervical spinal cord. Axial imaging demonstrates cord flattening by the anteriorly located neurenteric cyst hyperintense on both T1-weighted (c) and T2-weighted (d) MRI

The archetypal appearance of neurenteric cyst structure on diagnostic imaging is that of a lobulated homogenous mass without an associated mural nodule. Paolini *et al* described a unique instance of a false mural nodule identified within a neurenteric cyst.[42] The supposed nonenhancing nodule was determined to be a collection of mucinous debris positioned along the inferior aspect of the cystic chamber. Heterogeneous fluid content is an exceedingly rare finding with neurenteric cysts, but is nonetheless a possible confounding factor in diagnosis. The absence of a mural nodule can assist the clinician in differentiating between neurenteric cysts from other pathology such as neoplasms (e.g., neuroblastoma) and teratomas. The majority of neurenteric cysts are located ventral to the spinal cord.[11,13,18] There is often associated flattening, widening, and/or thinning of the spinal cord secondary to mass effect that is best appreciated on axial MRI sequences.[31,39] Over 90% of spinal neurenteric cysts are intradural and extramedullary, and several studies have shown that approximately 50% of identified spinal lesions are located specifically within the cervical spine, with most other cases occurring equally between the thoracic spine and thoracolumbar junction [Figure 3].[4,5,7,8,22,23,31,43] Less than 5% of lesions are located within the intramedullary compartment, mainly occurring in the upper cervical and lower thoracic spine.[5]

The relationship of neurenteric cyst incidence in tandem with osseous malformation warrants plain film and/or CT imaging to fully delineate the pathologic spectrum of this disease process. A case series of 23 patients with neurenteric cysts reported by Garg *et al* demonstrated that 87% of patients with vertebral anomalies evident on radiographic evaluation presented with neurenteric cysts at the same level as the bony abnormality.[25] Consequently, a thorough evaluation of vertebral anatomy is critical in developing a comprehensive treatment plan for neurenteric cysts.

## SURGICAL MANAGEMENT

Surgical resection is the first-line treatment for neurenteric cysts with the goal of gross total resection. When possible, total excision is the ideal outcome given the association between partial resection and cyst recurrence.[29] However, vertebral anomalies or extensive adhesion to neuronal anatomy can make complete resection difficult and hazardous.[13,25] Current literature lacks a consensus on the most appropriate surgical approach among the three prevailing methods: posterior, anterior, and lateral. Each poses potential benefits and risks associated with the treatment of the neurenteric cyst.

The posterior technique is the most widely reported surgical approach. Although neurenteric cysts typically present in a ventral position, the posterior approach is associated with fewer intraoperative complications.[24] Furthermore, the procedure can produce adequate access to the mass after cord manipulation and cyst aspiration. Risks associated with this approach are primarily linked to the typical complications associated with laminectomy such as spinal cord, dural, and nerve root injury. Additionally, hematoma may result from the posterior approach secondary to epidural venous hemorrhage or failure to control muscle bleeding. Posterior resection and the frequent need to aspirate cyst contents for this approach may carry a greater risk of membrane rupture causing cyst leakage potentially leading to meningeal irritation and meningitis.[11] Beyond complications, other factors limiting the use of a posterior surgical approach include the possibility that the spinal cord will obscure the view of the cystic structure and dissections of adhesions can prove difficult.[44]

The anterior approach has also been reported as an effective option for the resection of neurenteric cysts. While effective, this approach provides additional surgical complexity and an increased need for instrumented fusion as compared to the posterior approach. The anterior approach is associated with risks such as damage to adjacent neurovascular anatomy, fusion failure, hematoma formation, and CSF leakage. However, given the goal of total excision, the procedure's plane of access is advantageous in the resection of ventrally localized, extramedullary cysts.[11,29] Furthermore, the anterior approach may reduce the risk for cyst membrane rupture, thereby preventing leakage of contents.

The lateral approach is the least frequently reported technique for resecting neurenteric cysts. Associated risks are not dissimilar from the aforementioned methods such as durotomy and vascular damage. However, the technique provides an adequate view of the cyst-cord boundary while affording maximal preservation of normal anatomy.[19,44]

While consensus is absent with respect to the preferred approach, the literature does offer guidance regarding total versus partial resection in the case of intramedullary versus extramedullary cysts. As previously mentioned, the intradural/extramedullary location is the typical presentation accounting for approximately 95% of reported cases, whereas intramedullary neurenteric cysts comprise less than 5% of lesions.[31] More important than the relative rates of incidence is the fact that a clear plane of cleavage between the cyst and spinal cord is typically observed with extramedullary cysts only. Conversely, removal of intramedullary cysts is made more difficult due to the absence of a clear dissection plane. Consequently, the ability to completely resect intramedullary neurenteric cysts is difficult without an increased risk of neurological injury. Therefore, formidable adhesion to neuronal structures likely warrants partial resection.[5,31,40,45] In cases of intramedullary cysts, reported treatment has included cyst aspiration, partial resection with cyst marsupilization, and cystosubarachnoid shunting. Aspiration alone has been linked to recurrence and is, therefore, the least desirable intervention.[29] The utility of marsupilization and cystosubarachnoid shunting remains controversial. Several investigators cite cyst content leakage and associated CNS irritation as evidence to avoid these techniques.[45]

## SURGICAL OUTCOMES

Surgical treatment, particularly total resection, is most often curative of the sensory and motor deficits associated with neurenteric cysts. As described above, the risk of surgical morbidity must be considered when assessing the goals of surgical resection. Worsening of symptoms is documented in 11% of cases and failure to regain premorbid neurological function is cited in 18%.[24] The most frequently reported suboptimal outcome is cyst recurrence. Due to the rarity of neurenteric cysts, long-term follow-up is lacking amidst the available literature making true estimates of recurrence difficult.

Reports of postsurgical recurrence have ranged between 0% and 37%. Kim *et al*. and Cai *et al*. observed no recurrence in their case series of eight and seven patients, respectively.[31,46] Holmes *et al*. observed recurrence in only 4% patients.[32] Chavda *et al*. reported the longest follow-up at 30 years and observed a 37% recurrence rate among eight patients. Of note, all cases of recurrence in this study were seen in patients who underwent partial surgical resection.[47]

While the rarity of neurenteric cysts and its varying presentation does not allow for a definitive range of recurrence, the literature does establish a link between partial resection and recurrence. Kimura *et al* reviewed 18 recurrent cyst cases. In 16 of the cases, only a partial cyst resection was achieved.[13] Paleologos *et al*. reported a case series in which recurrence was absent in those patients who underwent gross total excision versus 11% in patients who underwent partial excision.[35] A similar trend was reported by Garg *et al*. No recurrence was observed in those patients with gross total resection, whereas recurrence was seen in five out of eight (63%) patients with partial resection.[25] Partial resection remains the primary risk factor for the recurrence in neurenteric cyst management as Menezes *et al* reported no correlation between recurrence and other factors such as age, sex, cyst size, and level.[29,48] Consequently, the surgeon must have the primary treatment goal of gross total excision of the cyst contents and cyst wall while appreciating the possible morbidity associated with achieving such a resection.

## CONCLUSIONS

Neurenteric cysts represent a small percentage of spinal disease clinically germane to the neurological surgeon. These lesions display characteristic histopathology including well-differentiated columnar or cuboidal epithelium with or without cilia and mucus globules. Patients presenting with these heterotopic lesions of endodermal origin often display symptomology consistent with compression of the spinal cord and associated nerve roots. MRI is the gold standard for characterizing neurenteric cysts. However, CT plays an important role in defining bony abnormalities that are often coexistent with displaced remnants of the developing gastrointestinal/respiratory tract. A complete surgical resection is the goal of the treatment. However, thoughtful consideration must be given to the potential benefits and risks associated with gross total resection in situations of neuronal adhesions or ambiguous dissection planes. Although outcome analyses of patients treated surgically for neurenteric cysts are limited, the data available demonstrate excellent neurologic recovery with nominal associated morbidity following resection. With a recurrence rate as high as 37%, patients with partial resection in particular should be followed with serial imaging to assess for re-accumulation of cyst contents.
